# Fault location of cable hybrid transmission lines based on energy attenuation characteristics of traveling waves

**DOI:** 10.1038/s41598-022-25976-8

**Published:** 2022-12-27

**Authors:** Wen Huo, Zhenbing Qu, Zirong Ao, Yongjun Zhang, Erleng Zhao, Chen Zhang, Hao Jiang

**Affiliations:** 1 Zhunneng Power Supply Company, Shenhua Group Zhungeer Energy Co. Ltd, Ordos, 010300 China; 2 School of Computer Science and Technology, China University of Mining and Technology, Xuzhou, 221116 China; 3 School of Electrical Engineering, China University of Mining and Technology, Xuzhou, 221116 China

**Keywords:** Electrical and electronic engineering, Energy grids and networks, Power distribution

## Abstract

The overhead-cable hybrid transmission lines are alternately connected by two types of lines, with a more complex structure and higher difficulty in fault location. This paper presents an ac-curate fault location method for overhead-cable hybrid lines based on traveling wave energy. Firstly, the basic concept of traveling wave energy is defined. Based on the attenuation charac-teristics of the traveling wave, the mapping relationship between traveling wave energy and fault location is analyzed. Secondly, considering the influence of S-transform error on the traveling wave energy propagation law, the traveling wave energy attenuation characteristics of common A-type and B-type hybrid lines are analyzed. Then, for the overhead-cable hybrid lines with different structures, the mapping relationship between the traveling wave energy at both ends of the line and the fault distance is quantitatively derived, and an accurate fault location method based on the initial traveling wave energy ratio at the same frequency at both ends of the line is proposed. Finally, a 110 kV hybrid transmission line fault simulation model is built in PSCAD/EMTDC, and the faults under different conditions are simulated in different line sections. The effectiveness and robustness of the proposed method are verified through the simulation.

## Introduction

Under the trends of urbanization, constructing the transmission lines is inevitably contradict to the development of towns and cities. Transmission lines are gradually evolving from a single overhead transmission line to a mixed transmission line of overhead lines and cable lines^1^. Statistics shows that single-phase grounding fault accounts for more than 80% of overhead line faults, and single-phase core and sheath faults account for a larger proportion of cable line faults. It is vital to improve the reliability of the distribution system to quickly locate the fault point of the cable mixed line after the fault occurs, troubleshoot and eliminate the fault, and restore the normal operation of the line as soon as possible^2^. Therefore, it is of great significance to find a fast and efficient fault location method.

To this end, common overhead line-cable hybrid lines can be divided into two different structures, type A and type B^3^. Type A lines consist of two parts of the line, one for the cable line and the other for the overhead line; Type B lines consist of three parts of the line, two for the overhead line and one for the cable line, and the cable line is located in the middle of the overhead line. From the structural perspective, there are significant differences between the overhead-cable hybrid line and the homogeneous line. Two following problems need to be considered in fault location: (1) the wave impedance at the cable connection point is discontinuous (2) the traveling wave will be refracted and reflected between the fault point, cable connection point and line end point. The propagation process is complex, which makes it a challenge to determine the source of the traveling wave head; The parameters of overhead lines and cable lines are different, and the propagation speed of fault traveling wave in the two types of lines is also different. The above problems make it difficult for the proposed fault location method for homogeneous transmission lines to be directly applied to hybrid transmission lines.

At present, the mainstream transmission line fault location methods include impedance method and traveling wave method^4^. Many scholars use impedance method to realize fault location for mixed cable lines. The literature^5^ mainly uses the power frequency electrical quantities at both ends of the line after the fault and the parameters of overhead lines and cable lines to calculate the voltage at the cable connection points, and determines the fault location through amplitude comparison. However, fault location of cable hybrid lines based on impedance method is greatly affected by transition resistance, load current and system impedance on the opposite side, which makes it difficult to further improve fault location accuracy in principle^6^.

The transient traveling wave method has been widely studied because of its simple principle and the advantages of not being affected by fault type and system operation mode, line asymmetry, etc. Literature^7–9^ proposed some innovative traveling wave fault location methods to solve the problem of inconsistent wave velocity between cable lines and overhead lines. Reference^10^ proposed a new traveling wave positioning method, defined the fault section decision function by using the difference between the forward propagation wave of DC current at both ends of the line and the difference between the reverse propagation wave, and then determined the fault location by using the different values of the decision function when the fault occurs in different line sections. Literature^11^ defines the node specific coefficient Q according to the amplitude of traveling wave, and proposes a new fault location principle by comparing the Q values of node nodes and fault points. Document^12^ proposed a positioning method based on support vector machine (SVM), which uses discrete wavelet transform (DWT) to extract fault transient information from measured voltage, and then uses wavelet coefficients of aviation mode voltage to achieve fault location. However, the principle of this method is relatively complex, and is greatly affected by the given length error of the double ended line and the time synchronization error of the double ended line, so it is difficult to achieve accurate fault location.

Based on the traveling wave method of cable, hybrid line fault location is mainly dedicated to solving the difficulties caused by inconsistent line wave speed. The traditional two-terminal fault location method is improved. However, in principle, the fault location method based on the arrival time of the traveling wave must be affected by the synchronization of the measurement equipment and the accuracy of the traveling wave speed, both of which are uncontrollable, and their errors will affect the location accuracy^13^. Therefore, the transmission characteristics of the fault traveling waves in the cable hybrid line need to be further studied, and the time–frequency domain characteristics of the transient fault traveling wave signal at the measurement point need to be explored^14^.

Due to the continuous development of fault location technology in recent years, various advanced intelligent algorithms emerge one after another, and various signal processing methods are also changing with each passing day. Many novel fault location methods for cable hybrid lines emerge. Literature^15^ proposes a fault location method based on Short Term Memory (LSTM) network, which uses LSTM network to adaptively learn input and output samples on the basis of the original wavelet theory, The LSTM fault location model is obtained, and then the fault location is carried out. Reference^16^ designed a transmission line fault location and protection scheme using Stockwell transform (ST), Wigner distribution function (WDF) and dissimilation factor (ACF). ST, WDF and ACF are used to analyze current signals, respectively calculate the Stockwell Failure Index (SFI), Wigner Failure Index (WFI) and Alienation Factor Failure Index (ACFI), and use them to derive the Hybrid Signal Processing Failure Index (HSPFI) to detect transmission line failures. Literature^17^ proposed a method based on the application of morphological gradient (MG) to the modal components of the current synchronously measured at both ends of the hybrid line to detect the transient components generated by the fault and locate the fault. Literature^18^ first defined two voltage symbolic functions, and then located the fault section according to the different numerical combinations of the two voltage symbolic functions. Then, combined the adaptive characteristic scale decomposition with the improved general local frequency decomposition, adaptively extracted the fault characteristic components, established the location equation within the section based on the characteristic components, and accurately located the fault. However, the above methods have high requirements for the accuracy of fault signals, weak processing capacity for interference signals, and high costs when put into practical application, making it difficult to be practical.

This paper analyses the changing characteristics of traveling wave energy with the help of advanced time–frequency analysis tools. Based on the attenuation characteristics of traveling wave energy when it is transmitted on cables and overhead lines, the traveling wave energy loss is used to describe the change of traveling wave at the discontinuity point of crossing wave impedance. It only needs to extract the initial traveling wave head of the fault, and does not need the support of the synchronization system, nor does it need to obtain the traveling wave velocity, to avoid the impact of synchronization error and wave velocity change on fault location in high-voltage long-distance transmission. The attenuation characteristics of travelling wave energy are used to more accurately characterize the changing pattern of travelling waves, and on this basis, further research into fault pinpointing methods for cable hybrid transmission lines is carried out. The rest of the paper is divided into five parts: “Analysis of fault traveling wave energy propagation characteristics” section analyses the attenuation characteristics of travelling wave energy and the mapping relationship between travelling wave energy and fault location. “Precise positioning of hybrid cable circuits” section investigates the hybrid line fault pinpointing algorithm based on the attenuation of travelling wave energy. “Simulation verification” section simulates to verify the effectiveness of the method. Finally, “Conclusion” section gives the conclusion.

## Analysis of fault traveling wave energy propagation characteristics

### Energy decay characteristics

**Figure 1:**
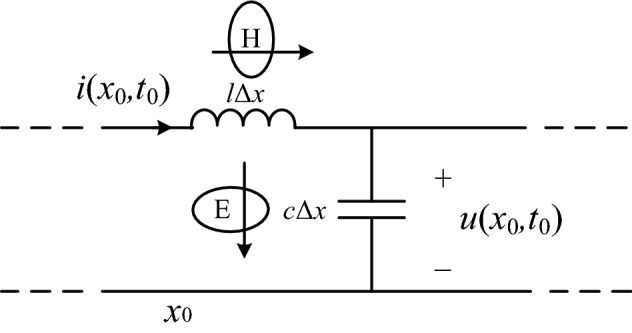
Schematic diagram of traveling wave energy propagation.

As shown in Fig. 1, when the traveling wave reaches the place, the electric field energy and magnetic field energy will be generated on the line micro-element *Δx* as in Eq. (1) where at the moment *t*_0_, the voltage and current at *x*_0_ on the line are *u*(*x*_0_*,t*_0_), *i*(*x*_0_*,t*_0_) respectively. *l* and *c* are the inductance and capacitance to ground per unit length of the line respectively.

1 $$W_{u} = \frac{1}{2}c\Delta xu(x,t_{0} ,\omega_{0} )^{2} ,\quad W_{i} = \frac{1}{2}l\Delta xi(x,t_{0} ,\omega_{0} )^{2}$$

The traveling wave voltage and current satisfy the relation:

2 $$u\left( {x,t,\omega_{0} } \right) = \sqrt{\frac{l}{c}} i\left( {x,t,\omega_{0} } \right)$$

Bringing Eq. (2) into either Eq. (1) shows that the electric field energy stored on the line is essentially the same as the magnetic field energy. Thus, the electromagnetic energy *W*_*x*_ in the line micro-element is:

3 $$W_{x} = W_{u} + W_{i} = cu(x,t_{0} ,\omega_{0} )^{2} \Delta x = li(x,t_{0} ,\omega_{0} )^{2} \Delta x$$

The electromagnetic energy *W*_*t*_ per unit time at* x*_**0**_ on the line is:

4 $$\begin{aligned} W_{t} & = cu(x_{0} ,t,\omega_{0} )^{2} v\Delta t = li(x_{0} ,t,\omega_{0} )^{2} v\Delta t = cu(x_{0} ,t,\omega_{0} )^{2} \Delta t\frac{1}{{\sqrt {lc} }} \\ & \quad u(x_{0} ,t,\omega_{0} )^{2} \sqrt{\frac{c}{l}} \Delta t = i(x_{0} ,t,\omega_{0} )^{2} \sqrt{\frac{l}{c}} \Delta t = u\left( {x_{0} ,t,\omega_{0} } \right)i\left( {x_{0} ,t,\omega_{0} } \right)\Delta t \\ \end{aligned}$$

For point *x*_0_ on the line, the traveling wave energy passing through the point at time *t*_1_ to time *t*_2_ can be expressed as follows:

5 $$W = \mathop \smallint \nolimits_{{t_{1} }}^{{t_{2} }} u\left( {x_{0} ,t,\omega_{0} } \right)i\left( {x_{0} ,t,\omega_{0} } \right)dt = \mathop \smallint \nolimits_{{t_{1} }}^{{t_{2} }} u^{2} \left( {x_{0} ,t,\omega_{0} } \right)\sqrt{\frac{c}{l}} dt = \mathop \smallint \nolimits_{{t_{1} }}^{{t_{2} }} i^{2} \left( {x_{0} ,t,\omega_{0} } \right)\sqrt{\frac{l}{c}} dt$$

Meanwhile, the power loss on the line will lead to the attenuation of traveling wave energy, and its value is:

6 $$\begin{aligned} P_{loss} & = i(x_{0} ,t_{0} ,\omega_{0} )^{2} R\Delta x + u(x_{0} ,t_{0} ,\omega_{0} )^{2} G\Delta x \\ & \quad = i(x_{0} ,t_{0} ,\omega_{0} )^{2} \left( {R + lG/c} \right)\Delta x \\ \end{aligned}$$

When the traveling wave with power *P* = *i*(*x*_0_,*t*_0_)^2^*Z* propagates on the line element d*x*, the power variation is Δ*P* = 2*Zi*(*x*_0_,*t*_0_)d*i,* due to the existence of R and G. Where, Z = R + jX, R is the line resistance, and X is the line reactance. Since the energy is decaying, the Δ*P* sign is negative, and in connection with Eq. (6) it is obtained that:

7 $$i(x_{0} ,t_{0} ,\omega_{0} )^{2} \left( {R + lG/c} \right)dx = - 2i\left( {x_{0} ,t_{0} ,\omega_{0} } \right)Zdi$$

By solving the differential equation about current and distance in Eq. (7), the following equation can be obtained:

8 $$i\left( {x,t,\omega_{0} } \right) = i\left( {x_{0} ,t_{0} ,\omega_{0} } \right)e^{{ - \frac{1}{2}[R/Z + lG/cZ]x}} = i\left( {x_{0} ,t_{0} ,\omega_{0} } \right)e^{ - \gamma x}$$

where γ is the propagation coefficient of traveling wave energy, which is related to the resistance, capacitance, inductance and conductance parameters of the line. Substitute Eq. (8) into Eq. (5) to obtain the propagation formula of traveling wave energy:

9 $$W\left( {x,t,\omega_{0} } \right) = i^{2} \left( {x_{0} ,t_{0} ,\omega_{0} } \right)Ze^{ - [R/Z + lG/cZ]x} = W\left( {x_{0} ,t_{0} ,\omega_{0} } \right)e^{ - 2\gamma x}$$

Equation (9) is the attenuation law of traveling wave energy derived from the power loss of resistance. The equation shows that traveling wave energy also decays exponentially during transmission. Combining with the frequency-dependent characteristics of the line, the propagation of traveling wave energy has the following characteristics: The high frequency traveling wave component has a large attenuation coefficient, and the traveling wave energy decays quickly in the propagation process. Correspondingly, the low frequency traveling wave component has a small attenuation coefficient, and the traveling wave energy decays slowly.

### Mapping between energy and fault location

According to the attenuation characteristics of travelling wave energy mentioned above, it can be seen qualitatively that the resistance and conductance of transmission lines will cause the reduction of travelling wave energy, and the longer the propagation distance, the smaller the value of travelling wave energy. Quantitatively, the attenuation of traveling wave signals conforms to the law of exponential attenuation, and the magnitude of traveling wave energy and the distance of traveling wave propagation can be described by an exponential function. Based on the above analysis, the mapping relationship between travelling wave energy and fault location at both ends of a single type of line is derived as follows:

Mathematically, the following differential equation can be used to describe the reduction of traveling wave energy during propagation, where A refers to the value of traveling wave energy and λ to the decay constant of traveling wave energy.

10 $$\frac{dA}{{dx}} = - \lambda A$$

One solution of the above equation is:

11 $$A\left( x \right) = A_{0} e^{ - \lambda x}$$

where *A*(*x*) represents the value of traveling wave energy at the distance *x* from the starting point, and *A*_0_ is the initial value of traveling wave energy.

**Figure 2:**
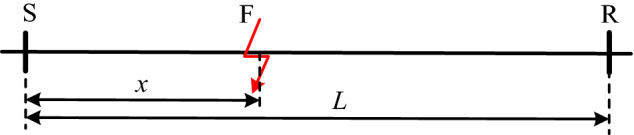
Uniform line fault diagram.

For the homogenized line shown in Fig. 2, the first and last ends of the line are noted as S and R. Assuming that the distance of the fault point from the S end of the line is x and the total length of the line is L, the energy of the traveling wave component at any frequency at the S and R ends of the line, which can be calculated by Eq. (12) :

12 $$\begin{aligned} W_{S} (\omega ) & = W_{F} (\omega )e^{ - \alpha (\omega )x} \\ W_{R} (\omega ) & = W_{F} (\omega )e^{ - \alpha (\omega )(L - x)} \\ \end{aligned}$$

In the formula, *W*_*S*_(*ω*) and *W*_*R*_(*ω*) are respectively the energy of the traveling wave component with the frequency of *ω* at the S-end and R-end of the line, *W*_*F*_(*ω*) is the energy of the initial traveling wave component with the frequency of *ω* at the fault point. Since it is a uniform line, the initial traveling wave energy propagated to both ends of the line is consistent, and α(ω) is the energy attenuation coefficient of the traveling wave component with the frequency of *ω*.

By dividing the upper and lower equations of Eq. (12), the energy of the unknown initial travelling wave of the fault *W*_*F*_(*ω*) is eliminated, and the following equation can be obtained:

13 $$\ln \frac{{W_{S} (\omega )}}{{W_{R} (\omega )}} = \alpha (\omega )(L - x) - \alpha (\omega )x$$

By moving ***x*** in Eq. (13) to the left of the equation, the relationship between the fault location and travelling wave energy at both ends of the line can be obtained:

14 $$x = \frac{1}{2}\left[ {L - \frac{1}{\alpha (\omega )}\ln \frac{{W_{S} (\omega )}}{{W_{R} (\omega )}}} \right]$$

The formula is the frequency ***ω***, based on the traveling wave energy attenuation theory derived from the fault location calculation formula, from the formula can be seen, the line length *L* is a known quantity, traveling wave energy attenuation coefficient *α*(*ω*) can be calculated through the line parameters, as long as the line can be obtained at both ends of the traveling wave energy *W*_*S*_(*ω*)*, W*_*R*_(*ω*), you can directly calculate the fault location.

### Error analysis of line wave amplitude extraction based on S-transform

The analysis of the mapping relationship between energy and faults is carried out under ideal conditions. It is considered that the traveling wave component of a single frequency can be extracted from the initial traveling wave head. However, due to the limitation of the current time–frequency analysis algorithm, it is impossible to accurately extract the signal of a specific frequency in the multi-frequency aliasing signal. For the traveling wave signal with a continuous spectrum, a certain frequency component after decomposition still contains other frequency components. Therefore, under the same fault, when the time–frequency analysis algorithm is used to decompose the measured fault traveling wave signals at different locations and obtain the traveling wave energy of a certain frequency, the calculated results will deviate from the real value of the descending wave energy of this frequency, which leads to the following two problems: first, the attenuation coefficient and refraction coefficient of traveling wave energy cannot be calculated by using line parameters; Second, the mathematical relationship between the measured value of traveling wave energy and the propagation distance no longer strictly satisfies the form of the exponential function.

In this case, the errors generated by the decomposition of traveling wave signals will definitely affect the positioning accuracy of the fault location algorithm based on Formula (14). Compared with uniform lines, the traveling wave energy changes on the non-fault section should also be considered for the cable mixed lines, and the correction of S-transform error should be paid more attention to during fault location. In theory, a mathematical formula can be used to describe the S-transform error and the relationship between the traveling wave propagation distance, eliminating the influence of S-transform on the positioning accuracy and fault traveling wave. However, one frequency component size is unknown, can't solve the S-transform error and its relationship with the propagation distance is fitting, but from another point of view, Fitting this relation is to describe the variation law of traveling wave energy propagating along the road more accurately. By fitting the relation between traveling wave energy attenuation coefficient α(ω) and traveling wave propagation distance x, the relation between S-transform error and traveling wave propagation distance can be indirectly reflected, and the accurate solution of traveling wave energy at the fault point can also be realized. Therefore, it is necessary to analyze and study the traveling wave energy variation rule under the effect of S-transform error according to the structural characteristics of cable mixed lines, so as to improve the accuracy of the fault location.

**Figure 3:**
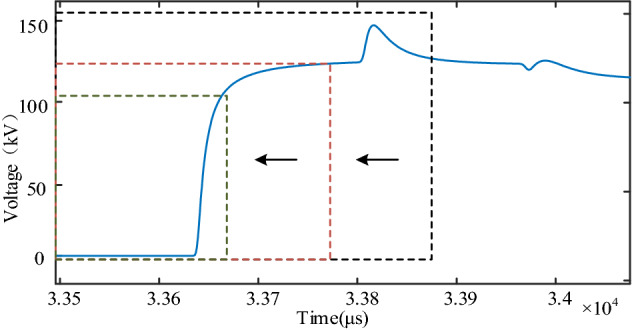
S-transform time window movement diagram.

As the analysis in this paper is directed at the initial travelling wave of the fault, fault information needs to be extracted from the initial traveling wave head, so the selection of fault data needs to be discussed before analyzing the effect of the S-transform error on the travelling wave energy change pattern. Figure 3 is the initial traveling wave diagram of voltage measured 50 km away from the fault point when the overhead line is faulty. The arrival time of traveling wave is 0.033636 s, and the arrival time of the first reflected wave is 0.033803 s. Below, the S-transform algorithm is used to decompose the signal in the time–frequency domain, and the change characteristics of the S-transform results of each frequency signal component are compared when the fault signals at different starting and ending times are used. The length of the S-transform time window in this paper is chosen to be 2 ms, with a total of 2000 time points. The S-transform time window is continuously moved from right to left to reduce the signal length of the effective waveform of the fault line waveform, and the S-transform results for the fault voltage signal are shown in Table 1.

**Table 1:** S-transform results of signals at different start and end times.

Time window		Frequency		
Start point	End point	20 kHz	50 kHz	80 kHz
31,842	33,842	43.5444	8.90061	1.269766
31,802	33,802	43.5444	8.90061	1.269766
31,782	33,782	43.5444	8.90061	1.269766
31,722	33,722	–	8.89214	1.269766
31,702	33,702	–	8.58569	1.269766
31,682	33,682	–	5.82425	1.268488

Table1 shows that the waveform data in the first row of the time window contains the initial travelling wave and reflected travelling wave, while the second row of time window only contains the initial travelling wave data. Comparing the S-transform results of the three frequencies, the results are completely the same. Since both ends of the line are completely reflected, the reflection coefficients are equal and cancel each other, so the reflected wave has no effect on the extraction of the initial traveling wave component. Comparison table of the same frequency of S-transform results under different time window, when the time to move out of the window to the left, the length of the fault effectively reduced, the transformation results gradually becomes poor, comparing the signals of different frequency, the greater the impact on the low frequency of signal, sometimes even unable to extract low frequency signal amplitude. As shown in Table 1, when the extraction frequency is 20 kHz, the fourth to sixth rows cannot display data, which means that with the left shift of the time window, the S-transform cannot extract amplitude at low frequency. Considering the attenuation characteristics of traveling wave, the components of traveling wave with higher frequencies decay quickly and are not easy to detect, while the frequencies with too low are easy to be aliased together and not easy to distinguish. Three frequencies of 20 kHz, 50 kHz and 80 kHz are selected from small to large, and the extraction effects of S transform under the three frequencies are compared and analyzed, and the results are shown in Table 1. The traveling wave component with 80 kHz frequency can obtain better decomposition effect. It can be seen that the faulty high-frequency components are concentrated in the front of the traveling wave head, that is, the part where the wave head rises quickly. The flatter part of the wave head has less influence on the extraction results of high frequency components. Therefore, when solving the traveling wave energy, it is necessary to ensure that the complete wave head of the initial traveling wave of the fault is contained in the S-transform time window.

## Precise positioning of hybrid cable circuits

### Classification of traveling wave energy variation laws

From the structure of the type A and type B hybrid lines and the location of the fault occurrence, the propagation path of the initial traveling wave of the fault can be divided into five categories as shown in Table 2.

**Table 2:** Different categories of initial traveling wave path.

Type	Propagation path
1	Fault point—Overhead line—Bus bar
2	Fault point—Cable line—Bus bar
3	Fault point—Overhead line—Cable connection point—Cable line—Bus bar
4	Fault point—Cable line—Cable connection point—Overhead line—Bus bar
5	Fault point—Overhead line—Cable connection point—Cable line—Cable connection point—Overhead line—Bus bar

Comparing the similarities and differences of the five types of propagation paths, the variation in traveling wave energy from the fault point to both ends of the line can be classified as a combination of the following six types of laws:

① Overhead line fault, the variation law of traveling wave energy on this line.
② Cable line fault, the variation law of traveling wave energy on this line.
③ The variation law of traveling wave energy before and after the cable connection point.
④ Overhead line fault, the variation law of traveling wave energy on the cable line directly connected to the fault line.
⑤ Cable line fault, the variation law of traveling wave energy on the cable line directly connected to the fault line.
⑥ Overhead line fault of the B-type hybrid line, variation law of traveling wave energy on another section of overhead line.

As long as the above-mentioned variation laws are clarified, the fault point data can be deduced from the data at both ends of the line to realize fault location.

### Determination of the attenuation coefficient

The B-type hybrid line simulation model is built in PSCAD to analyze and fit the variation law of traveling wave energy under different scenarios. The S-transform time window selected in this section is consistent with the previous paper. At the same time, considering the attenuation characteristics of traveling waves, the traveling wave components of higher frequencies decay quickly and are not easy to detect, while those of too low frequencies are easily mixed and not easy to distinguish. Therefore, the traveling wave components of 80 kHz frequency are used to obtain a better decomposition effect.

#### Overhead line fault, the variation of traveling wave energy in this section

**Figure 4:**
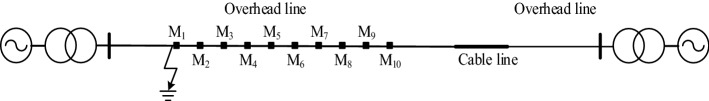
Simulation model of traveling wave energy variation of overhead lines (scenario 1).

**Figure 5:**
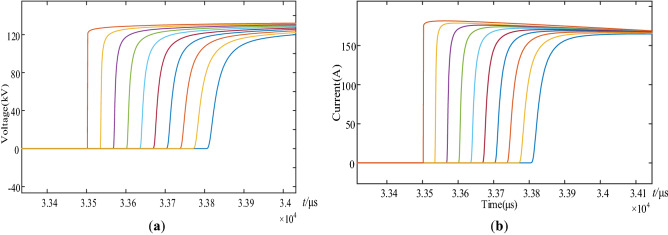
(**a**) Initial traveling wave waveform of voltage at each measuring point; (**b**) Initial traveling wave waveform of current at each measuring point.

Set a traveling wave measuring point every 10 km in the overhead line section of the simulation model. There are 10 points in total, the sampling frequency is 1 MHz, and the traveling wave measuring points are named M1 ~ M10 respectively. As shown in Fig. 4, an A-phase grounding fault is set on the left side of the measuring point M1, and the fault resistance is 10Ω, and the voltage and current waveforms at each measuring point are recorded. As shown in Fig. 5a,b from left to right show the initial traveling wave waveforms of voltage and current at M1–M10 respectively.

**Figure 6:**
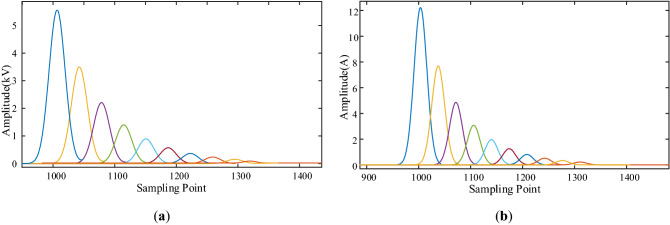
(**a**) S-transform results of voltage traveling wave at each measuring point; (**b**) S-transform results of current traveling wave at each measuring point.

In this paper, the S-transform is used to process the voltage and current traveling waves at the measuring points M_1_–M_10_. We calculate the modulus of each element in the complex matrix. Since the frequency of 80 kHz not only ensures the smoothness of the curve, but also reduces the influence of stray waves, so extract the frequency component of 80 kHz, and the results are shown in Fig. 6. The amplitude in Fig. 6a represents the voltage amplitude of 10 measurement points after undergoing S transformation, and the amplitude in (b) represents the current amplitude of 10 measurement points after undergoing S transformation. The nonlinear arc fault is considered in this paper. In the figure, from left to right are the S-transform results of traveling wave waveforms at M_1_–M_10_. Next, the traveling wave energy at each measurement point is calculated according to Eq. (15), where *W*_M*i*_ represents the traveling wave energy at measurement point M_*i*_, and *S*_*U*_*(d)* and *S*_*I*_*(d)* are the values of *d*-column of the voltage and current traveling wave S-transformation mode matrix, respectively. And *D*_*i*_ is the column number corresponding to the maximum value of the faulty traveling wave S-transformation waveform at M_*i*_. Therefore *S*_*U*_*(D*_*i*_*)* and *S*_*I*_*(D*_*i*_*)* are used to characterize the amplitude of 80 kHz frequency component, and n characterizes the duration of the 80 kHz frequency component. For n, in the actual simulation, we set n = 5, n = 10 and n = 20 respectively, and observe the influence of different n values on the processing results of S transformation. It is found that when n is 5, the fitting results of S transformation are not smooth enough. With the increase of n, the curve is gradually smoothed, and when n is 10, the fitting degree of the curve has met the requirements. If n is too large, the required computing power and time will also be greatly increased, and the cost is too high. Therefore, after comprehensive consideration, n = 10 is finally selected.

15 $$W_{Mi} = nS_{U} (D_{i} )S_{I} (D_{i} )$$

The results of the calculations are shown in Table 3:

**Table 3:** Traveling wave energy at different measuring points of an overhead line (scenario 1).

Measuring point	M1	M2	M3	M4	M5
Energy	1359.0	554.58	238.15	104.76	46.873

Measuring point	M6	M7	M8	M9	M10
Energy	21.238	9.591	4.258	1.9105	0.8718

Substituting the traveling wave energy measured at M_2_–M_10_ into the following formula to calculate the traveling wave energy attenuation coefficient at each position. The results are shown in Table 4.

16 $$\alpha_{1} (L_{{M_{1} M_{i} }} ) = - \frac{1}{{L_{{M_{1} M_{i} }} }} \ln \frac{{W_{{M_{i} }} }}{{W_{{M_{1} }} }}$$

**Table 4:** Traveling wave energy attenuation coefficients at different propagation distances (scenario 1).

Distance/km	10	20	30	40
Attenuation coefficient	8.9628 × 10^–5^	8.7079 × 10^–5^	8.5428 × 10^–5^	8.4176 × 10^–5^

Distance/km	60	70	80	90
Attenuation coefficient	8.2561 × 10^–5^	8.2367 × 10^–5^	8.2089 × 10^–5^	8.1686 × 10^–5^

Using the cubic function in MATLAB to fit the relationship between the energy attenuation coefficient and the propagation distance of the traveling wave, the results are as follows:

17 $$\begin{aligned} \alpha_{{1}} (x_{{1}} ) & = - {1}{\text{.846}} \times {10}^{{ - 20}} x_{{1}}^{{3}} { + 4}{\text{.291}} \times {10}^{{ - 15}} x_{{1}}^{{2}} \\ & \quad - {3}{\text{.596}} \times {10}^{{ - 10}} x_{{1}} { + 9}{\text{.279}} \times {10}^{{ - 5}} \\ \end{aligned}$$

where *x*_1_ is the distance from the head end to the fault point.

#### Cable line fault, the variation of traveling wave energy in this section

**Figure 7:**
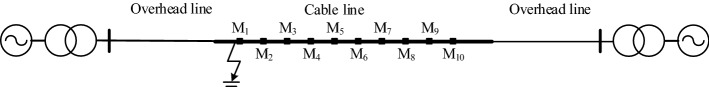
Simulation model of traveling wave energy changes of cable lines (scenario 2).

Referring to Fig. 7, set 10 traveling wave measuring points as M_1_–M_10_, and the interval of which is 2 km. Then setting the A-phase core sheath fault on the left side of the measuring point M1, and the fault resistance is 10Ω. The cable used in this section is the most common high voltage cable: cross-linked polyethylene cable, which is laid horizontally and directly buried. The length of the cable is more than 1 km, so the metal sheath of the cable is often cross-connected. According to formula (15) and formula (16), the traveling wave energy and attenuation coefficient are calculated respectively, and the cubic function is used for fitting. The calculated data and fitting results are shown in Tables 5 and 6:

**Table 5:** Traveling wave energy at different measuring points on the cable line (Scenario 2).

Measuring point	M1	M2	M3	M4	M5
Energy	1744.9	1579.6	1439.7	1321.4	1221.3

Measuring point	M6	M7	M8	M9	M10
Energy	1139.0	1068.4	1013.9	965.13	907.51

**Table 6:** Traveling wave energy attenuation coefficient for cable lines with different propagation distances (scenario 2).

Distance/km	2	4	6	8	10
Attenuation coefficient	4.9760 × 10^–5^	4.8068 × 10^–5^	4.6336 × 10^–5^	4.4594 × 10^–5^	4.2655 × 10^–5^

Distance/km	12	14	16	18	
Attenuation coefficient	4.0877 × 10^–5^	3.8778 × 10^–5^	3.7011 × 10^–5^	3.6319 × 10^–5^	

The fitting formula of the traveling wave energy attenuation coefficient and the propagation distance of the cable line and the schematic diagram of the fitting curve are as follows, where *x*_1_ is the distance from the fault point:

18 $$\begin{aligned} \alpha_{{2}} (x_{{1}} ) & = {2}{\text{.202}} \times 10^{{ - 18}} x_{{1}}^{{3}} - {6}{\text{.101}} \times 10^{{ - 14}} x_{{1}}^{2} \\ & \quad - {4}{\text{.261}} \times 10^{{ - 10}} x_{{1}} { + 5}{\text{.073}} \times 10^{{ - {5}}} \\ \end{aligned}$$

The remaining three types of propagation paths can be adaptive to various situations using the same method, and finally get: *α*_1_ ~ *α*_5_. ① $\alpha_{1} (x_{1} )$ is the attenuation coefficient of traveling wave energy under the scenario of overhead line fault. ② $\alpha_{2} (x_{1} )$ is the attenuation coefficient of traveling wave energy under the scenario of cable line fault. ③ $\alpha_{3} (x_{1} ,x_{2} )$ is the attenuation coefficient of traveling wave energy on the cable line directly connected to the fault overhead line. ④ $\alpha_{4} (x_{1} ,x_{3} )$ is the attenuation coefficient of traveling wave energy on the overhead line directly connected to the fault cable line. ⑤ $\alpha_{5} (x_{1} ,x_{2} ,x_{3} )$ is the attenuation coefficient of traveling wave energy on right overhead line under the scenario of B-type overhead line fault.

Due to space limitations, please refer to the Appendix for specific expressions. Among them, *x*_1_ represents the propagation distance of the fault traveling wave on the fault line. *x*_2_ represents the length of the cable line adjacent to the fault line. *x*_3_ represents the length of the overhead line adjacent to the fault line. Where the cable connection points need to consider the commutation factor, and the simulation takes the average value as the refraction factor of the traveling wave energy in the fault location algorithm. Considering A, B-type mixed connected line structure, there are three cases: The refraction coefficient *γ*_11_ takes 0.26615 when the traveling wave enters the overhead line side from the cable side under the scenario of overhead line fault. The refraction coefficient *γ*_12_ takes 0.26601 when the traveling wave enters the overhead line side from the cable side under the scenario of cable line fault. The refraction coefficient *γ*_2_ takes 0.26623 when the traveling wave enters the cable line side from the overhead line side.

Compared with the uniform line, the fault location of the line-cable hybrid line is more complicated: From the point of view of the line structure, the existence of line-cable connection points makes the traveling wave energy decrease step by step, and different line types introduce additional traveling wave energy attenuation coefficient. From the influence of S-transform error, it is not only necessary to consider the change of the traveling wave energy attenuation coefficient on the faulty section, but also to accurately describe the change of the attenuation coefficient on the non-faulty section. Based on the above two reasons, it is necessary to modify the homogeneous line fault location method deduced above to make it suitable for precise location of line-cable hybrid line faults.

### Precise positioning method

**Figure 8:**
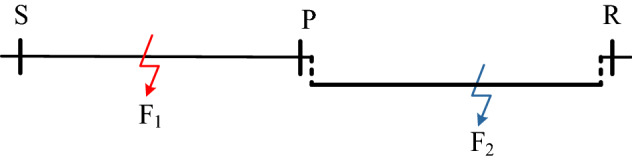
Schematic diagram of type A cable hybrid transmission line.

As shown in Fig. 8, the length of overhead line SP is *L*_SP_, and the length of cable line PR is *L*_PR_. The attenuation coefficients of the traveling wave energy at the fault distance *x*_1_ for the traveling wave component with frequency *ω* on the overhead line and the cable line as the fault section are *α*_1*ω*_(*x*_1_) and *α*_2*ω*_(*x*_1_) respectively. The attenuation coefficients of the traveling wave energy at the fault distance *x*_1_ for the traveling wave component with frequency *ω* on the overhead line and the cable line as the non fault section are *α*_3*ω*_(*x*_1_,*x*_2_) and *α*_4*ω*_(*x*_1_,*x*_3_) respectively. Since the lengths of overhead line and cable line × 2 and × 3 are determined values, *α*_3*ω*_(*x*_1_,*x*_2_) and *α*_4*ω*_(*x*_1_,*x*_3_) can be reduced to *α*_3*ω*_(*x*_1_) and *α*_4*ω*_(*x*_1_).

In addition, the special fault location of the cable connection point has been judged in the fault section location, so it is not analyzed below.

For overhead line fault F1, the initial traveling wave energy with frequency ω propagated from the fault point to both ends of the line is *W*_F_(*ω*). When the traveling wave propagates to the S end of the line and the cable connection point P, the traveling wave energy at these two places can be calculated by the following formula:

19 $$\begin{aligned} W_{{\text{S}}} (\omega ) & = W_{{\text{F}}} (\omega )e^{{ - \alpha_{{{1}\omega }} (x)x}} \\ W_{{{\text{Pf}}}} (\omega ) & = W_{{\text{F}}} (\omega )e^{{ - \alpha_{{{1}\omega }} (L_{{{\text{SP}}}} - x)(L_{{{\text{SP}}}} - x)}} \\ \end{aligned}$$

In the formula, *x* represents the distance from the S end of the line. When the traveling wave propagates from the point P to the R end of the line, the traveling wave energy at the R end can be calculated by the following formula:

20 $$W_{{\text{R}}} (\omega ) = \gamma_{{2}} (\omega )W_{{{\text{Pf}}}} (\omega )e^{{ - \alpha_{{{3}\omega }} (L_{{{\text{SP}}}} - x)L_{{{\text{PR}}}} }}$$

In the formula, γ2(ω) represents the traveling wave energy refraction coefficient of the traveling wave component with frequency ω when it passes from the overhead line side to the cable side. The meaning of this coefficient is shown in formula (8), where *W*_Pf_(*ω*) and *W*_Pb_(*ω*) respectively represent the traveling wave energy of the traveling wave component with frequency *ω* before and after passing through the cable connection P-point.

21 $$\gamma_{{2}} (\omega ) = \frac{{W_{{{\text{Pb}}}} (\omega )}}{{W_{{{\text{Pf}}}} (\omega )}}$$

Similarly, by dividing the two equations of Eq. (19), the energy *W*_F_(*ω*) of the unknown traveling wave when initial fault is eliminated. And then combining Eqs. (20) and (21), a mathematical relation only about *W*_S_(*ω*) and *W*_R_(*ω*) with fault distance *x*_1_ can be obtained as follows:

22 $$\begin{aligned} \ln \left[ {\gamma_{{2}} (\omega )\frac{{W_{{\text{S}}} (\omega )}}{{W_{{\text{R}}} (\omega )}}} \right] & { = }\alpha_{{{1}\omega }} (L_{{{\text{SP}}}} - x)(L_{{{\text{SP}}}} - x) \\ & \quad - \alpha_{{{1}\omega }} (x)x + \alpha_{{{3}\omega }} (L_{{{\text{SP}}}} - x)L_{{{\text{PR}}}} \\ \end{aligned}$$

From the above equation, it can be seen that *W*_S_(*ω*), *W*_R_(*ω*) and *L*_SP_ in the expression of *W*_P_(*ω*) are known quantities or can be obtained by actual measurement. And the variation law of *γ*_2_(*ω*), *α*_1*ω*_(*x*_1_), *α*_3*ω*_(*x*_1_) has been obtained by fitting. In identifying the fault is located in the overhead line section, the fault location can be calculated based on the traveling wave energy relationship between the S and R ends of the line shown in Eq. (22) to achieve the precise location of the overhead line fault on the A-type cable hybrid line.

Similarly, the fault location formula of the cable line can be obtained as:

23 $$\begin{aligned} \ln \left[ {\frac{1}{{\gamma_{{{11}}} (\omega )}}\frac{{W_{{\text{S}}} (\omega )}}{{W_{{\text{R}}} (\omega )}}} \right] & { = }\alpha_{{{2}\omega }} (x)x - \alpha_{{{2}\omega }} (L_{{{\text{PR}}}} - x)(L_{{{\text{PR}}}} - x) \\ & \quad - \alpha_{{{4}\omega }} (L_{{{\text{PR}}}} - x)L_{{{\text{PR}}}} \\ \end{aligned}$$

**Figure 9:**
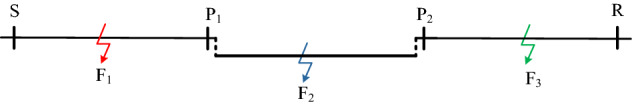
Schematic diagram of type B cable hybrid transmission line.

Similarly, the expression of the mapping relationship between traveling wave energy and fault location can be derived for B-type hybrid line faults with different fault locations. As shown in Fig. 9, the length of the overhead lines SP_1_ and P_2_R are *L*_SP1_ and *L*_P2R_ respectively, and the length of the cable P_1_P_2_ is *L*_P1P2_. When a fault occurs on the overhead line SP_1_/P_2_R, the traveling wave energy attenuation coefficient of the traveling wave component with the frequency *ω* on the SP_1_/P_2_R section at the fault distance *x*_1_ is *α*_1*ω*_(*x*_1_). And the traveling wave energy attenuation coefficient of the traveling wave component with the frequency *ω* on the P_2_/P_1_ point at the fault distance *x*_1_ is *α*_3*ω*_(*x*_1_). Also, the traveling wave energy attenuation coefficient of the traveling wave component with the frequency *ω* on the R-end/S-end at the fault distance *x*_1_ is *α*_5*ω*_(*x*_1_). When a fault occurs on the cable line P_1_P_2_, the traveling wave energy attenuation coefficient of the traveling wave component with the frequency *ω* on the P_1_P_2_ section at the fault distance *x*_1_ is *α*_2*ω*_(*x*_1_). And the traveling wave energy attenuation coefficient of the traveling wave component with the frequency *ω* on the R-end/S-end at the fault distance *x*_1_ is *α*_4*ω*_(*x*_1_).

For overhead line fault F_1_, when the initial traveling wave with the frequency *ω* propagates to the S-end and R-end of the line, the traveling wave energy is as follows:

24 $$\begin{aligned} W_{{\text{R}}} (\omega ) & = \gamma_{{{12}}} (\omega )\gamma_{{2}} (\omega )W_{{\text{F}}} (\omega )a_{2} \\ a_{2} & = e^{{ - \alpha_{{{1}\omega }} (L_{{{\text{SP}}_{1} }} - x)(L_{{{\text{SP}}_{1} }} - x)}} e^{{ - \alpha_{{{3}\omega }} (L_{{{\text{SP}}_{1} }} - x)L_{{_{{{\text{P}}_{1} {\text{P}}_{{2}} }} }} }} e^{{ - \alpha_{{{5}\omega }} (L_{{{\text{SP}}_{1} }} - x)L_{{_{{{\text{P}}_{{2}} {\text{R}}}} }} }} \\ W_{{\text{S}}} (\omega ) & = W_{{\text{F}}} (\omega )e^{{ - \alpha_{{{1}\omega }} (x)x}} \\ \end{aligned}$$

Dividing the two formulas in formula (24) and the result is as follows:

25 $$\left\{ {\begin{array}{*{20}l} \begin{aligned} \ln \left[ {\gamma_{{{12}}} (\omega )\gamma_{{2}} (\omega )\frac{{W_{{\text{S}}} (\omega )}}{{W_{{\text{R}}} (\omega )}}} \right] & = \alpha_{{{1}\omega }} (L_{{{\text{SP}}_{1} }} - x)(L_{{{\text{SP}}_{1} }} - x) \\ & \quad - \alpha_{{{1}\omega }} (x)x + \ln C \\ \end{aligned} \hfill \\ {\ln C = \alpha_{{{3}\omega }} (L_{{{\text{SP}}_{1} }} - x)L_{{{\text{P}}_{1} {\text{P}}_{2} }} + \alpha_{{{5}\omega }} (L_{{{\text{SP}}_{1} }} - x)L_{{{\text{P}}_{2} {\text{R}}}} } \hfill \\ \end{array} } \right.$$

In the formula, *x* represents the distance between the fault point F_1_ and the S-end of the line, and *γ*_12_(*ω*) represents the traveling wave energy refraction coefficient when the traveling wave component with frequency *ω* passes from the cable side to the overhead line side under the overhead line fault.

For cable line fault F_2_, when the initial traveling wave with the frequency *ω* propagates to the S-end and R-end of the line, the traveling wave energy is as follows:

26 $$\begin{aligned} W_{{\text{R}}} (\omega ) & = \gamma_{{{11}}} (\omega )W_{{\text{F}}} (\omega )a{}_{1} \\ a_{1} & = e^{{ - \alpha_{{{2}\omega }} (L_{{_{{{\text{P}}_{1} {\text{P}}_{{2}} }} }} - x)(L_{{_{{{\text{P}}_{1} {\text{P}}_{{2}} }} }} - x)}} e^{{ - \alpha_{{{4}\omega }} (L_{{_{{{\text{P}}_{1} {\text{P}}_{{2}} }} }} - x)L_{{_{{{\text{P}}_{{2}} {\text{R}}}} }} }} \\ W_{{\text{S}}} (\omega ) & = \gamma_{{{11}}} (\omega )W_{{\text{F}}} (\omega )e^{{ - \alpha_{{{2}\omega }} (x)x}} e^{{ - \alpha_{{{4}\omega }} (x)L_{{_{{{\text{SP}}_{1} }} }} }} \\ \end{aligned}$$

Dividing the two formulas in formula (26) and the result is as follows:

27 $$\left\{ {\begin{array}{*{20}l} \begin{aligned} \ln \left[ {\frac{{W_{{\text{S}}} (\omega )}}{{W_{{\text{R}}} (\omega )}}} \right] & { = }\ln C - \alpha_{{{2}\omega }} (x)x \\ & \quad + \alpha_{{{2}\omega }} (L_{{{\text{P}}_{1} {\text{P}}_{2} }} - x)(L_{{{\text{P}}_{1} {\text{P}}_{2} }} - x) \\ \end{aligned} \hfill \\ {\ln C = \alpha_{{{4}\omega }} (L_{{{\text{P}}_{1} {\text{P}}_{2} }} - x)L_{{{\text{P}}_{2} {\text{R}}}} - \alpha_{{{4}\omega }} (x)L_{{{\text{SP}}_{1} }} } \hfill \\ \end{array} } \right.$$

In the formula, *x* represents the distance between the fault point F_2_ and P_1_ point. For overhead line fault F_3_, when the initial traveling wave with the frequency *ω* propagates to the S-end and R-end of the line, the traveling wave energy is as follows:

28 $$\begin{aligned} W_{{\text{R}}} (\omega ) & = W_{{\text{F}}} (\omega )e^{{ - \alpha_{{{1}\omega }} (x)x}} \\ W_{{\text{S}}} (\omega ) & = \gamma_{{{12}}} (\omega )\gamma_{{2}} (\omega )W_{{\text{F}}} (\omega )a_{3} \\ a_{3} & = e^{{ - \alpha_{{{1}\omega }} (L_{{{\text{P}}_{{2}} {\text{R}}}} - x)(L_{{{\text{P}}_{{2}} {\text{R}}}} - x)}} e^{{ - \alpha_{{{3}\omega }} (L_{{{\text{P}}_{{2}} {\text{R}}}} - x)L_{{_{{{\text{P}}_{{1}} {\text{P}}_{{2}} }} }} }} e^{{ - \alpha_{{{5}\omega }} (L_{{{\text{P}}_{{2}} {\text{R}}}} - x)L_{{_{{{\text{SP}}_{{1}} }} }} }} \\ \end{aligned}$$

Dividing the two formulas in formula (28) and the result is as follows:

29 $$\left\{ {\begin{array}{*{20}l} \begin{aligned} \ln \left[ {\frac{{1}}{{\gamma_{{{12}}} (\omega )\gamma_{{2}} (\omega )}}\frac{{W_{{\text{S}}} (\omega )}}{{W_{{\text{R}}} (\omega )}}} \right] & = - \alpha_{{{1}\omega }} (L_{{{\text{P}}_{{2}} {\text{R}}}} - x)(L_{{{\text{P}}_{{2}} {\text{R}}}} - x) \\ & \quad { + }\alpha_{{{1}\omega }} (x)x - \ln C \\ \end{aligned} \hfill \\ {\ln C = \alpha_{{{3}\omega }} (L_{{{\text{P}}_{{2}} {\text{R}}}} - x)L_{{{\text{P}}_{{1}} {\text{P}}_{{2}} }} + \alpha_{{{5}\omega }} (L_{{{\text{P}}_{{2}} {\text{R}}}} - x)L_{{{\text{SP}}_{1} }} } \hfill \\ \end{array} } \right.$$

In the formula, x represents the distance between the fault point F3 and R-end of the line.

## Simulation verification

### Precise positioning simulation

The line in the simulation adopts the B-type connection mode. The total length of the transmission line is 112 km, of which the length of the two overhead lines is 60 km and 40 km respectively, and the length of the cable line is 12 km. The segment location method described in the literature is used to locate the segment firstly, and then the precise fault location is solved through the loop iteration of the attenuation coefficient and the virtual fault point.

**Figure 10:**
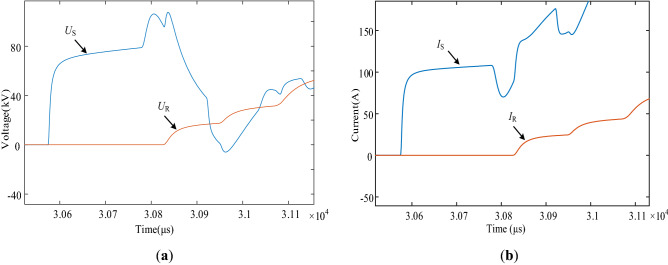
(**a**) Voltage traveling wave waveform at S-end and R-end of the line; (**b**) Current traveling wave waveform at S-end and R-end of the line.

Firstly, the overhead line fault is used as a case study to demonstrate the fault location process. We set an A-phase ground fault at 22 km from the S-end of the line, and then measure initial fault traveling wave at both ends of the line. The waveform is shown in Fig. 10.

**Figure 11:**
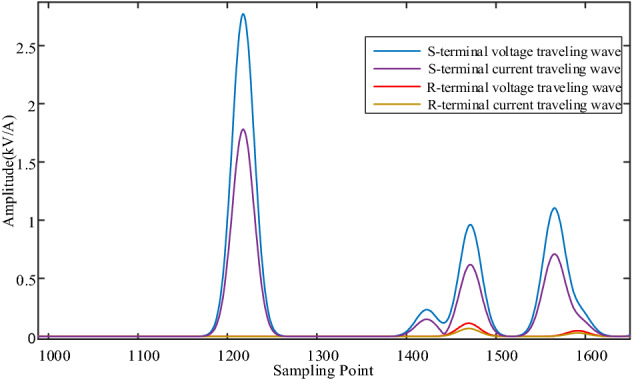
S-transformation results of voltage and current traveling waves across the line.

**Figure 12:**
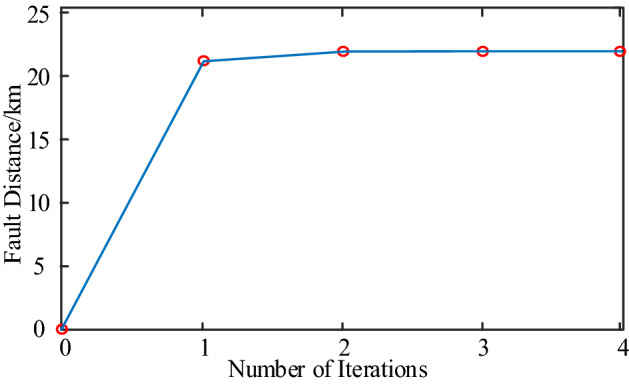
Convergence of iterative algorithms.

Using the S-transform to extract the 80 kHz traveling wave component, the S-transform results are shown in Fig. 11. The amplitude in Fig. 11 represents the amplitude results of the voltage and current at the initial and end of the line after S transformation. And calculating the traveling wave energy according to the method described in “Determination of the attenuation coefficient” section, the traveling wave energy at the S-end and R-end of the line is 472.227 and 0.216063 respectively. The iteration of the ranging algorithm is implemented in MATLAB by combining the variation law of the traveling wave energy attenuation coefficient of the overhead line and the Eq. (25). The results of each iteration are plotted in Fig. 12, from which the computational results gradually converge with the increase of the number of iterations, and the algorithm converges quickly. After 4 iterations, the difference between the two adjacent computed fault distances is 0.001 km, which satisfies the requirement of Δx = 0.001 km ≤ 10^−3^ km, and the iterative process ends. The fault location result is 21.779 km, which is only 0.221 km different from the real fault distance, so the proposed algorithm has good location accuracy.

### Sensitivity analysis

To verify the reliability and robustness of the algorithm, the structure, length, fault initial phase angle and fault transition resistance of the line are changed respectively. The positioning precision in the above scenarios is analyzed and compared with the positioning effect of the uncorrected method, and the results are shown in Tables 7 and 8. The uncorrected method means that the same traveling wave attenuation coefficient is used to calculate the fault location without considering the influence of the S-transform error on the attenuation coefficient in different scenarios. The traveling wave attenuation coefficient is obtained by fitting the traveling wave energy measured along the line. The traveling wave attenuation coefficient of the overhead line is 8.7742 × 10^–5^, and of the cable line is 3.7334 × 10^–5^.

**Table 7:** Effect of initial phase angle of fault and transition resistance on positioning results.

Line structure	Fault distance (km)	Fault initial phase angle	Fault resistance (Ω)	Positioning results /km	Absolute positioning error /km
60–12–40 km	40	30°	10	39.713	0.287
			200	39.718	0.282
			300	39.721	0.279
		60°	10	39.714	0.286
			200	39.718	0.282
			300	39.721	0.279
		90°	10	39.714	0.286
			200	39.718	0.282
			300	39.720	0.280

**Table 8:** Location results under different line structures and fault locations.

Line structure	Fault distance (km)	Positioning results /km		Absolute positioning error /km
		Uncorrected method	Method of this paper	
50–15 km	15	15.508	14.872	0.128
	25	24.794	24.831	0.169
	35	34.410	35.140	0.140
	54	50.698	53.837	0.163
	58	55.015	58.170	0.170
	62	58.099	61.251	0.749
80–18 km	30	30.878	29.906	0.094
	40	40.259	40.217	0.217
	60	58.141	59.763	0.237
	86	79.694	85.771	0.229
	90	83.252	89.685	0.315
	95	88.046	94.840	0.160
60–12–40 km	25	27.006	25.283	0.283
	35	35.680	34.849	0.151
	65	63.020	65.237	0.237
	68	66.114	68.149	0.149
	85	83.912	85.255	0.255
	100	97.609	100.386	0.386
80–15–60 km	30	33.649	29.785	0.215
	50	51.446	49.805	0.195
	85	83.167	85.197	0.197
	90	87.977	90.154	0.154
	120	117.135	120.181	0.181
	135	130.117	134.709	0.291

The results in Table 7 show that the fault initial phase angle and the fault transition resistance variation have little effect on the positioning results. This is because the initial phase angle and the transition resistance only change the amplitude of the initial wave head of the fault and do not change the rising process of the wave head. That is, the S-transformation of the fault traveling waves under different conditions will only proportionally reduce the traveling wave energy of the voltage and current traveling waves. However, if the fault resistance is high enough, and the fault transient characteristics are weak, or the traveling wave signal has basically decayed and disappeared after long distance transmission, the initial traveling wave cannot be accurately detected, and the method cannot accurately locate the fault location.

From the positioning results in the Table 8, it can be conclude that in the uncorrected method has relatively large errors in the positioning results, especially in the case of long line lengths, and the positioning results cannot meet the actual engineering needs. The method in this paper considers the influence of S-transform on the extraction of the single frequency component of the traveling wave, and has a good localization effect on any point fault on the line.

The analysis and results from Fig. 3 and Table 1 show that the distortion of the waveform has a small effect on the S-transform results. Table 9 shows the location results of the constant resistance fault and arc fault under different fault locations. From the data in the table, it can be seen that the arc fault location error has increased compared with constant resistance faults. However, it can still meet the requirements of practical engineering applications.

**Table 9:** Positioning results under constant resistance fault and arc fault.

Line structure	Fault initial phase angle	Fault distance (km)	Fault type	Positioning results /km	Absolute positioning error /km
60–12–40 km	90°	40	Constant resistance fault	39.716	0.284
			Arc fault	40.649	0.649
		65	Constant resistance fault	65.240	0.240
			Arc fault	65.411	0.411
		85	Constant resistance fault	85.257	0.257
			Arc fault	84.524	0.476

## Conclusion

Based on the defects of the traditional localization method about traveling wave arrival time, this paper searches for the characteristic quantity that can be applied to fault localization from the perspective of traveling wave energy attenuation characteristics. And analyzing in detail the mapping relationship between the characteristic quantity and the fault location to achieve precise localization. In principle, this method is completed by using the difference of traveling wave energy at both ends of the line. It only needs to simply measure and calculate the fault traveling wave energy. In application, the method doesn’t need to ensure strict time synchronization between the measuring points, nor does it need to use the information of the fault reflection wave head, which reduces the introduction of errors and can achieve precise and reliable fault location.

## Supplementary Information

Supplementary Information.

## Data Availability

All data generated or analysed during this study are included in this article and its supplementary information file.
